# Selections and Abstracts

**Published:** 1914-03

**Authors:** 


### SELECTIONS AND ABSTRACTS

### THIRTY YEARS' PROGRESS IN OBSTETRICS.

By E. E. Montgomery, M. D.

PROFESSOR OF GYNECOLOGY IN THE JEFFERSON MEDICAL COLLEGE OF PHILADELPHIA.

It may seem presumptuous to some that I should attempt to present a paper upon this subject before a society which numbers so many men apparently better qualified by experience, but I would assure them that much of the work I have done in later years has been the result of obstetrical work.

I am further impelled to discuss it by the consideration that thirty years ago this month I read a paper before this society on the subject, "Is Craniotomy on the Living Child Justifiable?"

Cæsarian section was then considered a dernier ressort, and was practiced only when all other measures failed, or where it was evident that no other measures were practicable. It was made the operation of election only when the conjugata vera was 2 to 2 1-2 inches (5 to 6.25 cm.) or less, and the child alive. Some authorities asserted that it need not be employed where the smallest diameter measured 1 1-2 inches (3.75 cm.).

Some of the recommendations in the paper were: the examination of all pregnant women during the last month of gestation, and where the pelves were below normal measurements and the conjugata vera measured 2 1-2 inches (6.25 cm.) or over, the induction of premature labor.

Abortion was to be considered only in women pregnant for the first time, in whom it was evident a viable child could not be born.

Symphysiotomy, which had lately been revived, was advocated in women whose true conjugate measured 2 1-2 inches (6.25 cm.) or over, as affording a chance for the child.

If the physician has determined (as he should be able to do) that a living child cannot be delivered per vias naturales, he should proceed without delay to Cæsarian section. Every hour of delay increases the peril to mother and child. Were such the practice the craniotomist would stand on the same level as the abortionist.

The Porro operation was advocated where women had been long in labor.

Cæsarian section was then unpopular among English-speaking people, as is evident from the fact that craniotomy was employed in nearly one out of every two hundred cases. A larger percentage of mothers survived where delivery took place by the horn of a cow than where the patient was the victim of the surgeon's knife. Cæsarian section then had a maternal mortality of over 26 per cent, while the fetal mortality was 19 per cent.

The conclusions were as follows: Craniotomy is unjustifiable because: (1) It considers only the life of the mother and destroys the child, while it is our duty to save both. (2) In pelves with a contracted conjugate other alternatives are equally safe for the mother which afford the child a chance for life. These alternatives are suggested in the following order: where the conjugate measures 3.25 inches (8 cm.) or over, the forceps; 2.75 inches (7.5 cm.), version; 2.375 inches (5.9 cm.), symphysiotomy, followed if necessary, by the forceps. In all subsequent pregnancies, and in the first when distortion is discovered sufficiently early, premature labor should be induced. (3) In pelves measuring less than 2 1-2 inches (6.25 cm.) Cæsarian section affords better results for the mother, and should be done whether the child be living or dead. Operative interference should be early in all cases requiring it. The obstetrician should control events, not be controlled by them.

This paper was discussed by Albert H. Smith, Goodell, Parish, W. S. Stewart, and J. V. Kelly. Of these men the only survivor, Dr. Kelly, was the only one to sustain my views. The propositions were considered unduly radical. My statistics were questioned when I stated that the maternal mortality of craniotomy was 19 per cent. The only statistics quoted to controvert mine were by Parish, who stated that Ellerslie Wallace had lost but three mothers out of twenty-one cases of craniotomy, but this was 14 per cent, and in the hands of a teacher and an expert in obstetrics.

A review of the last thirty years' work displays the course strewn with worn-out theories, tried out and discarded procedures, and yet the course has ever been a progressive one. What would seem as radical yesterday is the conservative course today. A consideration of the progress comprises the study of the toxemias, which manifest their baneful influence in eclampsia and pernicious vomiting; the various forms of infection producing the many manifestations formerly comprised under the designation of puerperal fever; the vaccines and serums employed in its treatment; the influence upon the diagnosis and prognosis of hypotension and hypertension, and the indications produced thereby for the treatment of the affected individuals; the various procedures for the premature interruption of pregnancy, and the wide extension of operative work.

The progress covers every portion of the field of obstetrics —a compass too great for the limitations of this paper; so I shall content myself with consideration of that portion which may be said to be allied to my former paper, the conclusions of which form the introduction to this article. Many procedures which were considered justifiable measures for employment in suitable cases have been relegated to the dust-heap of oblivion. More and more, there has been an appreciation of the value of human life, so that the physician no longer stands as the executioner who sacrifices human life, but in the attitude of a savior who recognizes that in every case of labor he has two lives to consider and must exercise his ability to the utmost to give each a chance to live.

Abortion is no longer considered a justifiable alternative in contracted pelves, even if the latter precludes the delivery of a living child through the natural canal. Conditions do arise, however, in which interference with the process of gestation must be considered in the interest of the life of the mother. These measures have been classified by Rosner into three groups: First, the excitors to uterine contractions, as bougies, puncture of the ovum,separation of its lower pole, injections of glycerin and other agents, long-continued vaginal irrigation and electrization of the uterus. The second group comprises measures which combine contractions of the uterus with dilation of the cervix. Measures suitable to produce expulsion, as small dilating bags, graduated bougies, gauze tampon of the cavity and cervix, and dilatation with laminaria tents. The third group permits the dilatation of the cervix and the removal of the ovum. The procedures applicable to this group are removal of the ovum with the curette after dilatation with Hegar's bougies removal of the ovum after dilatation of the uterus with such instruments as that of Bossi, or after vaginal Cæsarian section.

Unfortunately the majority of these procedures afford no certainty as to the time of the completion of the evacuation. The procedure set in motion may vary from twenty to one hundred and sixty hours before its termination. In serious tubercular lesions of the lung, in non-compensated cardiac lesions, and in toxemias from serious renal disorders, speedy evacuation may be a very important factor. Between the tenth and twenty-fourth weeks, Rosner enthusiastically advocates vaginal Cæsarian section as possessing the following advantages: The termination of the procedure is no longer a question of uncertainty; it requires minutes rather than hours. The evacuation of the uterus can be complete. With an anesthetic the procedure is painless, and the subsequent convalescence undisturbed. The procedure is attended with no traumatism of the surrounding tissues, consisting of an incision in the anterior lip which can be sutured immediately following delivery, restoring the uterus to a normal condition. The procedure is almost bloodless. The earlier methods are not free from the danger of infection, for it has been demonstrated that the employment of elastic bags, the use of bougies, repeated digital examinations, are all attended by the introduction of agents which favor infection.

Many of these patients submit to the production of abortion with the greatest abhorrence and reluctance, and as hours or days are required for its completion they undergo a species of mortal torture. I have employed the procedure in but one case, and then when the patient regained consciousness after a short anesthesia the uterus had been emptied and the cervix resutured.

In two instances in early pregnancy in which an unsuspected pregnated ovum had been imperiled by intrauterine manipulation and conditions otherwise had demanded abdominal section, I have opened the fundus, removed the fetal sac, sponged out the uterine cavity, and sutured the uterine wall. One of these patients has since given birth to two children. The vaginal Cæsarian section is almost bloodless, while the slower methods of evacuating the uterus are at times attended by profuse bleeding.

A review of the modern obstetric literature cannot but impress one with the greater precision in determining the measurements of the pelvis; with the recognized importance of careful study of the condition of the pregnant woman in order that her attendant shall not overlook indications of serious toxemia, a cervical position of the placenta threatening hypertension, abnormal conditions of the pelvis, a disproportionate size of the fetus, and unfavorable positions and presentations.

The responsibility of the would-be attendant to know the condition of his patient and be able to safeguard not only her interests, but those of her child as well, is becoming more definitely recognized. In the light of more accurate knowledge of the condition of the patient it is not surprising to learn of the more prompt resort to the proper operative procedures in cases demanding them. The intelligent practitioner would consider it a reflection upon his skill and judgment to permit a woman with persistently high arterial tension, despite measures employed to promote elimination through the skin and kidneys, to continue until her life, or subsequent good health, is imperiled by eclampsia; to permit a woman with an evident cervical implantation of the placenta to lose her life, and that of her child, through a terrific hemorrhage; or to permit a woman with a recognized pelvic contraction to be subjected to various efforts at forcible delivery when it can be determined that such measures must prove unsuccessful.

It is not creditable to one's skill as an obstetrical engineer to subject a woman with a contracted pelvis, or who carries a child with a disproportionately large head, to prolonged tension with forceps, to find, what he should have known, that delivery per vias naturales is impossible. The operative field in obstetrics has been widely expanded during the years under consideration. New operative measures have been tried out; some have been discarded, and others have stood the test of time, but the wildest enthusiast for Cæsarian section can scarcely be otherwise than surprised at its adoption for conditions undreamed of thirty years ago. Instead of being the operation of last resort, as it then was, it has become the procedure of selection, and the life-saving results of such a course have exceeded our wildest dreams. Naturally there is still much diversity of opinion as to its adaptability to various conditions.

Some advise the delivery by Cæsarian section in all contracted pelves; in disproportionate size of the child, so that its head does not engage in the superior strait; in malpositions of the fetus, especially face presentations; in prolapse of the cord; in placenta previa; and in the arrest and prevention of eclampsia. Alternative procedures are the induction of premature labor, version, the application of forceps, vaginal Cæsarian section, and pubiotomy, hebosteotomy, and hebotomy, as it is variously designated. The Porro operation is still held in reserve for infected cases, and various modifications of the Cæsarian section to avoid the peritoneal cavity have been instituted. The test of the value of any obstetric procedure should be, not only its capacity to save the lives of both mother and child, but also its influence upon the subsequent life of the former.

The aim of every procedure should be to conserve health as well as life, and an operative procedure which endangers the woman, causes her to be subsequently handicapped and dooms her to semi-invalidism, must be to that degree condemned. An obstetric operative procedure, then, to be classed as acceptable today must afford the greatest safety to mother and child, the least danger of morbidity and consequent sequelæ. Naturally the method of delivery must ever depend upon the exigencies of the case, and in the choice and adaptation of the particular procedure lies the opportunity for the display of skill and good judgment upon the part of the medical attendant. His ability to recognize the abnormalities in the pelvis; disparities between the fetus and the canal through which it must pass; malpositions of the fetus and prompt measures for their correction; to distinguish danger-signals for the mother, as existing placenta previa, threatened eclampsia, exhaustion of her forces, protection from infection; and for the fetus, its inability to endure further prolongation of labor—all are indications of his skill as an obstetrician. In his enthusiasm for operative work he should not fail to realize that the great majority of women will still succeed in delivering themselves, and nature's method, whenever possible, should be utilized.

As it is impossible in advance to determine the size and molding qualities of the fetus, or the strength and force of the uterine contractions, it is advisable in moderate contraction to resort to the test of labor before instituting operative interference. In cases, however, in which it is evident that abnormality is such as to make interference imperative, the various adaptable procedures should be carefully considered.

The Induction of Premature Labor.—The first procedure which demands consideration in such a patient where the physician has studied conditions early in pregnancy, should be induction of premature labor. This procedure for the best interests of the fetus should not be performed before the thirty-fourth to thirty-fifth week of pregnancy, as statistics have demonstrated that 80 per cent of the children thus delivered prior to the thirty-fourth week succumb. The procedure when employed in women with pelves measuring less than 3 inches (7.5 cm.) is attended with such a frightful fetal mortality as to make its employment unjustifiable. Indeed, when we consider that the mortality for the fetus in pelves between 3 inches (7.5 cm.) and 3 1-2 inches (8.7 cm.) varies between 25 and 33

per cent, it would seem that induction of premature labor as an elective procedure for contracted pelves should be discarded.

Acute or chronic disorders may make the procedure important in the interest of either mother or child. In some of these conditions, as in valvular lesions of the heart, the strain of labor may be prejudicial, and the operation designated as vaginal Cæsarian section greatly lessens the danger. This procedure completely removes the resistance of the cervix and permits the prompt termination of labor by the application of forceps when the head is presenting.

Version: High Forceps.—Neither version nor high forceps are longer considered operations of election in contracted pelves owing to the high fetal mortality. Forceps can be employed to facilitate labor in cases in which additional force is needed, but the old procedure of the operator bracing himself for a long and hard pull is no longer considered in the interest of either mother or child. In contracted pelves the place of the forceps, according to Kerr, is where the measurement of the vera is between 3 1-2 and 3 1-4 inches.

Version was formerly considered advisable in that the application of the forceps anteroposterior to the child's head increased the lateral diameter, but it has been demonstrated that this is not true, the vertical diameter being lengthened instead. Experience has shown that when moderate traction by forcps is ineffective in advancing the labor it is safer, for mother and child, to afford more room by section of the pelvic bones.

Pubiotomy; Hebotomy; Hebosteotomy.—The operation of symphysiotomy, which thirty years ago I suggested as an alternative in a limited number of cases, has been practically discontinued, as injuries of the urethra and bladder were frequent, separation of the sacroiliac articulation occurred in the contracted cases, and the patient subsequently had unpleasant mobility between the pubic bones.

Pubiotomy, which has been substituted for it, consists in an incision through the pubic bone of one side with a chain saw. It may be done subcutaneously. The proceeding is of limited application. It should be confined to contractions of the vera between 3 inches (7.5 cm.) and 3.8 inches (9.5 cm.), and may be employed when it is evident that the patient cannot be delivered spontaneously, or the application of forceps has demonstrated that the birth can only occur after undue and dangerous traction. Such a head, however, can frequently be delivered by forceps after the increased space gained by pubiotomy.

The operation was devised by Gigli in 1893, and was first performed in this country by E. B. Montgomery, of Quincy, Ill. The situation of the incision to one side lessens the injury to the soft parts. The operation has been advocated by Morse for relief in cases of face presentation. He attributes to Dr. E. P. Davis the first suggestion of symphysiotomy as a means to promote delivery in face presentation.

Of the two hundred and ninety-four cases of pubiotomy collected by Doderlein, seventy-seven were treated by the open method, with a maternal mortality of 10 per cent. The remainder were by the subcutaneous procedure, with a maternal mortality of 4 per cent. Sarvey reported one hundred and twenty cases with a maternal mortality of 2.5 per cent, and a fetal mortality of 7 per cent, while in two hundred and sixteen cases of symphysiotomy the mortality was 12.8 per cent. The enlarged space secured by incision of the bone still leaves the soft parts subject to stretching and even tearing, so that the convalescence is delayed and the danger of infection promoted.

Up to May 10, 1910, twenty-five pubiotomies were performed in the obstetrical clinic of Johns Hopkins Hospital without maternal mortality, and with but three fetal deaths. As it is an operation done only for the child, a reasonable prospect for a living child should present to justify its performance.

Vaginal Caesarian Section.—Vaginal Caesarian section we have described as a method for rapidly completing abortion between the tenth and twenty-fourth weeks. The procedure has been widely heralded as a means to promote delivery, and relief of patients threatened with or suffering from eclampsia. Peterson has collected five hundred and thirty cases in which this procedure was performed in eclampsia, in which there were one hundred and twenty-four deaths of mothers—a mortality of

23.4 per cent—in the work of one hundred operators. Henry D. Fry says vaginal Caesarian section has done more to lessen the mortality of eclampsia than any other procedure. DeLee earnestly advocated early resort to operative interference in eclampsia, and quotes Winter as reporting that of twenty-nine patients treated before they had six convulsions not one died, while under a waiting policy the mortality was large. Of eclamptics treated expectantly with spontaneous labor, 40 per cent died; expectantly until the os dilated, 30 per cent mortality; with mild means to dilate the cervix, 25 per cent; and with cervical Caesarian section in thirty-four cases the mortality was 9 per cent. Where vaginal Caesarian section was done immediately after the first seizure in twenty-two cases there was no mortality. This procedure, however, is of no efficacy at term in contracted pelves, and is of doubtful expediency in primiparae.

Abdominal Caesarian Section.—The abdominal or classical Caesarian section has the advantage of being applicable to all cases demanding speedy delivery, whether the dystocia be due to pelvic deformity, disproportionate size of the fetus, malpositions and presentations, or cicatrical conditions in the soft; and has been resorted to for placenta previa, eclampsia, prolapsed cord, cancer of the cervix, cicatricial changes in the vagina, myomatous or malignant growths of the uterus, ovarian cysts situated in the birth canal, and other obstructive causes.

Thirty years ago I urged that the conditions demanding Caesarian section be recognized, and the operation where required be performed early. I insisted that if so practiced its percentage of mortality would not be greater than that of the operation then known as ovariotomy. Later practice has demonstrated this to be correct. Reynolds in an analysis of two hundred and eighty-nine cases by twenty different operators found it practicable to divide the cases of Caesarian section into three classes, which he calls primary, secondary, and late.

The primary included those subjected to operation prior to or at the beginning of labor, of which there were eighty-two cases, with but one death, a mortality of between 1 and 2 per cent.

Secondary, sections comprising those in which the operation followed a few hours' labor which had demonstrated the futility of the patient's efforts to deliver herself—one hundred and twenty-eight in number with six deaths, or a mortality of 3.8 per cent.

Late cases comprised forty-nine in which there had been an unduly long first stage, or an arrest of the head, and these furnished six deaths, or 12 per cent mortality. He had thirty cases in his own experience without any mortality.

Schauta had one hundred and twenty cases of classical Caesarian section, with but one death. Boyd reports twenty-seven cases with no maternal loss, and the death of one child which was dead before he began the operation. Macpherson reports thirty-nine cases of Caesarian section, of which thirty were performed for the second time, seven for the third time, and one each for the fourth and fifth times. In these cases there were three maternal deaths, one of which occurred before the beginning of the procedure, and one fetal death. Humpstone had twenty-five cases without mortality. Certainly I could not have had a more satisfactory confirmation of my prophecy.

The increasing mortality with the prolongation of labor makes it evident that this is due to infection. To prevent the baneful effects of infection various operators have endeavored to enter and empty the uterus without opening the peritoneal cavity. These were denominated the extraperitoneal Caesarian, and were performed through a lateral incision in one flank by Frank and Latzko. The abdomen opened to the peritoneum; the latter pushed up from the bladder until the under surface of the uterus was reached, and incised below the peritoneum, was suggested by Bumm. Hirst opens across the peritoneum, and sutures the parietal and uterine peritoneum. All these procedures are complicated, prolonged, and open up loose connective tissue capable of infection.

Freund quotes twenty-seven cases with no maternal mortality, some of which were women who had been in labor from six to twenty hours. He attributes the favorable result to the fact that the peritoneum had not been opened; but Humpstone had twenty-five of the classical Caesarian section, of which but six were elective, the remainder having been in labor from two to fifty hours, one of them having undergone repeated attempts to deliver with forceps, yet all recovered. In the light of such experience one does not seem justified to refuse delivery by Caesarian section even if the child's fetal heart sounds be feeble, for one cannot predetermine the fetal resistance.

The physician in attendance upon a case of confinement cannot be too strongly imbued with the thought that he is responsible for two lives, that no step shall be taken for the preservation of the one which unduly imperils the other. Actuated by such views he will have made a careful physical examination of the patient prior to the onset of labor in order to prepare himself for its particular exigencies. The responsible members of the family will have been informed of the possibilities confronting the delivery, so that no valuable time will be lost when the exigencies arise. The entire management of the case will be conducted with the most strenuous asepsis, that the life of the mother will not be unduly imperiled should extreme measures be demanded for the preservation of her offspring. Where conditions exist, such as marked deformity of the pelvis, the birth canal obstructed by cicatricial changes, by non-displaceable benign or malignant growths, threatened or existing eclamptic attacks, recognized implantation of the placenta in front of the fetus, or prolapse of the cord, no time will be lost in restorting to the proper remediable procedure.

Caesarian section has advantages over the alternative methods of delivery in that the operator can choose the time for the procedure, can govern the amount of traumatism, and in non-septic cases can feel that his patient will be free from subsequent morbidity and unfortunate sequelae. Reaching a case late when the patient's condition is such, and the history obtainable makes it evident that she has been subjected to severe and prolonged man handling, he will investigate the condition of the mother and her child, and where evidences of life are found in the latter will employ the measures which promise the most hopeful outlook for both. All experience has demonstrated that the earlier the Caesarian section can be utilized, and with the least previous manipulation, the less the mortality.

The procedure to avoid entrance to the peritoneal cavity do not altogether avert danger, for the infected uterus remains when the operation has been accomplished without entering the peritoneum. Experience shows that the peritoneum has been torn in 9 to 15 per cent of the cases. A greater amount of time is required in the more complicated procedure, and in the necessity for the frequent employment of instruments to complete delivery, and, as we have already asserted, a septic uterus remains. It would seem to me a wiser course in evidently infected cases to make the classical incision, carefully protect the peritoneal cavity by gauze packing, and complete the operation by means of the Porro procedure. The patient is then free from the dangers incident to an infected uterus.

Eclampsia.—Later years have presented a greatly extended employment of the Caesarian section, and an important one of these is in the treatment of eclamptic cases. Experience has demonstrated that the toxemias are arrested with the evacuation of the uterus. The toxic manifestations are associated with marked hypertension, and consequently the attacks are promoted and dangerous sequelae engendered by severe and prolonged efforts in delivery.

Measures have been employed to lessen the peril by doing vaginal Caesarian section, but even after the resistance of tne cervix has been overcome there still remains the undilated soft parts in the primipara, and the possibility of a narrowed pelvis, or a disproportionate size of the fetus which may neccessitate resort to further instrumental measures to facilitate delivery. No plan of management affords equal facility to Caesarian section in the freedom from irritability and the prompt evacuation of the uterus. The prudent physician will not wait for eclamptic seizures before resorting to it where he finds hypertension persists despite the remedial measures employed for its reduction.

Placenta Previa.—The cervical implantation of the placenta has been considered by many as an additional indication for Caesarian section, in that it affords the mother opportunity for escape from a serious dilemma equal to the chances presented by other measures, and greatly lessens the peril for her offspring.

Cragin says he has never seen Caesarian section indicated for uncomplicated placenta previa. In the Sloan Hospital, of forty-nine cases four mothers died, 8.1 per cent mortality, and an infant mortality of 51 per cent. Edger, who does not believe in Caesarian section for placenta previa, gives his maternal mortality as 7.5 per cent, fetal 32.25 per cent. Pankow advocates the operation, and cites twenty-three cases without maternal mortality, while in forty-nine women treated by other measures eight died. He is confident six of these could have been saved if abdominal Caesarian section had been promptly employed.

Prolapsed Cord.—The employment of Caesarian section for delivery where the cord is prolapsed may seem almost an unjustifiable extension of the operation, but with the almost insuperable difficulties to replacement of the umbilical cord, the head engaged, and with a fetal mortality of 50 per cent, it does not seem out of place that the most expeditious method of delivery should be employed. The prolapsed cord affords a ready method to determine the condition of the fetus. It would be worse than folly to do a Caesarian section when pulsations have ceased in the Cord.

Selected

---

### THE PHYSICIAN AND THE INCOME TAX.

By Arthur N. Taylor, LL. B., New York.
ATTORNEY AND COUNSELLOR AT LAW.

As we approach the end of the present month we realize, perhaps for the first time, that we are confronted by a new law, far-reaching and drastic in its provisions and replete with penalties, with which one must either promptly attempt to comply or, by omitting so to do, assume the liability of the penalty which will doubtless soon be meted out to those within its terms who have not complied. It is believed that nearly all desire to comply with the law, but with this desire come two questions, first, is one within the terms of the law and is any affirmative act on one's part necessary? and, second, if within its terms, how is this compliance to be met with?

All persons residing within the United States, and all citizens thereof residing abroad, having a net income of over $3,000 (or if married and living with their wives, a net income of over $4,000) are subject to the "normal" tax of 1 per cent on the excess of such exempt amount. The exemption of $4,000 is, however, not allowable to each husband and wife, but only the two together; thus, if the husband has a net income of $3,000, and the wife a net income of $2,000, they must pay a normal tax on $1,000. Nonresident aliens are also taxable upon the income from property in the United States or from any business, trade or profession carried on in the United States. In addition to the normal tax, "additional" tax is payable at graduated rates on the portion of one's income exceeding $20,000 per annum.

Partnerships are not required to account for their income unless requested by the Commissioner of Internal Revenue or a collector of internal revenue; the returns of the individuals composing the partnerships being considered sufficient to account for the income of the partnership.

In making up a schedule of one's income there must be included all income from salaries (except salaries of officers and employes of a State or political division thereof and of certain Federal officers), wages or compensation for personal services in whatever form, income and profits from trade, commerce, and from sales or dealing in property. It is not necessary to include, however, dividends from the stock or corporations subject to tax unless one's income with such dividends exceeds $20,000 a year, but it does not appear to be necessary to include unpaid accounts accruing within the year. Upon this question, we find it stated in the helpful little book of Mr. Arthur F. Speer, head of Corporation Tax Division, Internal Revenue Department, United States Treasury, as follows:

Persons receiving fees or emoluments for professional or other services, as in the case of physicians or lawyers, should include all actual receipts for services rendered in the year for which return is made, together with all unpaid accounts, charges for services, or contingent income due for that year, if the same are considered good and collectible. Should any such accounts prove to be bad or uncollectible, the amount of same are deductible from income for the year in which they are ascertained to be losses and are so treated and acknowledged by the taxable person. . . . Debts may be considered to be found worthless only after legal proceedings to recover the same have proved fruitless, or it is clearly evident that the debtor is insolvent and that proceedings to collect the debt would avail nothing.

When an account returned and taxed in one year is found in a subsequent year to be uncollectible, it appears that the amount of that account may be deducted from the income of such subsequent year. Against these items, one is entitled to deduct all interest paid within the year on indebtedness accruing within that year; the necessary expense of carrying on one's business, but not living or family expenses, that being included in the $3,000 or $4,000, as the case may be; all taxes paid within the year (assessments for local benefits not being included); losses during the year incurred in trade or arising from fires, storms, or shipwreck, and not compensated for by insurance or otherwise; debts due which are actually ascertained to be worthless and charged off within the year. There is also an allowance permitted for wear and tear of property from its use, exhaustion of mines, 5 per cent of the gross value of the output of the mine being deducted; and the amount of income upon which the tax has been withheld for payment at the source of the income.

If, after computing the items of one's income and subtracting all proper deductions, there remains a balance of over $3,000 or $4,000, as the case may be, one must then prepare, verify, and file one's return with the collector of internal revenues in the district in which one resides or has one's principal place of business, on or before the first day of March.

Blanks have been prepared showing so clearly and plainly the character of information required, and with such full instructions that apparently no difficulty should be experienced in filling them out. These blanks may be had from the collector in one's district. In making out the report for the year ending December 31, 1913, it must be borne in mind that the period covered commences on March 1, and is therefore calculated as five-sixths of a year only. If the income for this period exceeds $2,500, the person being unmarried or not living with his wife, then the excess of that amount is taxable, while the $4,000 annual exemption, for the ten month period becomes $3,333.33.

The penalties for failure to comply with the law are severe, and, if the rigorous enforcement of the penalties under the former corporation tax law may be regarded as a criterion from which to judge the future attitude of the Federal authorities under the present law, it would seem exceedingly unsafe to permit oneself to become liable to those penalties. The failure to have the return in the hands of the collector on or before March 1 renders one subject to a fine of from $20 to $1,000. For failure to make a return or to verify it, the commissioner is authorized and directed to add 50 per cent to the taxes found due. For making false or fraudulent return, the penalties prescribed are, a fine not exceeding $2,000, or imprisonment for not over one year, or both. The collector may also add 100 per cent to the tax. At any time within three years after the return became due, the commissioner may, upon discovery of its absence or errors, assess the tax which shall then immediately become due.

The commissioner makes the assessment of the tax payable and notifies the person on or before June 1, and the tax becomes payable to the collector on or before June 30. To any sums unpaid after June 30, and for ten days after notice and demand thereof by collector, there shall be added a penalty of 5 per cent, together with interest at the rate of 1 per cent a month upon the tax from the time it became due.

In case a physician feels that he has been erroneously assessed or unjustly taxed, he may either file a claim for abatement before the tax is paid or for a refund after the payment, asking for either the return of all or a portion of the tax paid as the circumstances require. Blanks have been prepared and will be furnished by the collector, together with full information correct return of all of the annual gains, profits, and income from both as to their preparation and the nature of evidence required to support the claim. These claims are also filed with the collector.

The conditions under which it is most likely to become necessary to file a claim either for abatement or refund, grow out of the provision of the law requiring the collections of taxes at its source. Under this head, the law provides that all firms, copartnerships, companies, corporations, joint stock companies, associations and insurance companies, in whatever capacity acting, including lessees or mortgagors of real or personal property, trustees acting in any trust capacity, executors, administrators, agents, receivers, conservators, employers, and all officers and employes of the United States Government having the control of receipt or payment of interest, rent, salaries, wages, premiums, annuities, compensation, remunerations, emoluments, or other fixed or determinable annual gains, profits and incomes exceeding $3,000 for the taxable year other than dividends of capital stock, are required to deduct the normal tax therefrom and pay it to the collector. In case the party from whose money this tax is deducted desires to avail himself of the additional exemptions, he should, within thirty days prior to the first day of March, file a notice with the person who is required to withhold and pay the tax, claiming the benefit of the exemption. If the exemption claimed is the additional $1,000 to which one married and living with his wife is entitled, then this fact should be properly set forth. If, on the other hand, exemptions are of the character of business expenses or losses as heretofore mentioned in this article, then the notice should be accompanied with a all other sources as well as the deductions asked for. This statement must also become a part of the return to be made by the person required to withhold and pay the tax.

The law also requires the withholding of the normal tax or 1 per cent from fixed and determinable annual or periodical gains or profits derived from interest on bonds and mortgages or deeds of trust or other similar obligations of corporations, joint stock companies and associations, and insurance companies regardless of the amount of such interest payment. This provision also includes the payment of coupons, checks, or bills of exchange, in payment of interest on bonds of foreign countries and foreign mortgages and like obligations, not payable in the United States. It is readily apparent that one entitled to such payment might be otherwise entirely exempt from the tax law, one's entire net income being less than $3,000. To gain the benefit of this exemption and secure the repayment to one of the tax deducted, it becomes necessary to prepare and file a claim for abatement of the character indicated above.

In addition to the normal tax, an "additional tax" is imposed according to the following schedule. For the portion of all incomes exceeing $20,000 a year and not exceeding $50,000 a year an additional tax of 1 per cent for the portion of incomes exceeding $50,000 a year and up to $75,000 a year an additional tax of 2 per cent for the portion of incomes exceeding $75,000 per year and up to $100,000 a year, an additional tax of 3 per cent; for all incomes exceeding $100,000 a year and up to $250,-000, an additional tax of 4 per cent; for all incomes exceeding $250,000 a year and up to $500,000 a year, an additional tax of 5 per cent, and for all incomes exceeding $500,000 an additional tax of 6 per cent.

Manifestly, we do not attempt to do more than give a brief outline of that phase of the law affecting the individual. The law has other features and imposes burdens upon individuals in other relations.—New York Medical Journal, January 24, 1914.

